# Gut microbiota derived metabolites in cardiovascular health and disease

**DOI:** 10.1007/s13238-018-0549-0

**Published:** 2018-05-03

**Authors:** Zeneng Wang, Yongzhong Zhao

**Affiliations:** Department of Cellular and Molecular Medicine, Lerner Research Institute, Cleveland Clinic, Cleveland, OH 44195 USA

**Keywords:** gut microbiota, metabolites, cardiovascular health, cardiovascular disease

## Abstract

Trillions of microbes inhabit the human gut, not only providing nutrients and energy to the host from the ingested food, but also producing metabolic bioactive signaling molecules to maintain health and elicit disease, such as cardiovascular disease (CVD). CVD is the leading cause of mortality worldwide. In this review, we presented gut microbiota derived metabolites involved in cardiovascular health and disease, including trimethylamine-*N*-oxide (TMAO), uremic toxins, short chain fatty acids (SCFAs), phytoestrogens, anthocyanins, bile acids and lipopolysaccharide. These gut microbiota derived metabolites play critical roles in maintaining a healthy cardiovascular function, and if dysregulated, potentially causally linked to CVD. A better understanding of the function and dynamics of gut microbiota derived metabolites holds great promise toward mechanistic predicative CVD biomarker discoveries and precise interventions.

**Electronic supplementary material:**

The online version of this article (10.1007/s13238-018-0549-0) contains supplementary material, which is available to authorized users.

## INTRODUCTION

There is a big gap in interpreting the molecular physiology by using the human genome coding capacity encompassing 23,000 coding genes (Gonzaga-Jauregui et al., 2012). The human gut is inhabited with 100 trillion microbes, with the majority as bacteria and archaea, fungi and microeukaryotes (Wampach et al., 2017). Almost 10 million coding genes of the microbiota have been uncovered, greatly expanding the coding capacity of our human as a superorganism (Qin et al., 2010; Li et al., 2014). Gut microbiota are essential to human health in many aspects, such as training intestinal epithelial barrier, modulating immuno-function, digesting host indigestible nutrients, producing vitamins and hormones and preventing pathogenic bacterium colonization (Schuijt et al., 2016). For a healthy subject, gut microbiota homeostasis is maintained with pathogenic microbe growth under control. Once the balance breaks, i.e., dysbiosis, pathogenic microbes thrive, leading to gut related diseases, such as inflammatory bowel disease (IBD), obesity, allergic disorders, diabetes mellitus, autism, colorectal cancer and cardiovascular disease (DeGruttola et al., 2016; Yang et al., 2015; Battson et al., 2017). Fecal microbiota transplantation has shown great efficacy in managing *Clostridium difficile* infection and Crohn’s disease (Bakken et al., 2013; Paasche 2013; Zhang et al., 2013). In animal model, fecal microbiota transplant to germ free mice recipients has been shown to transmit obesity and atherosclerosis susceptibility, suggesting the great potential of fecal microbiota transplantation in treating a panel of complex disease (Gregory et al., 2015; Turnbaugh et al., 2006). In addition, the prebiotic and probiotic administrations also show beneficial effects in optimizing gut microbiota community structure and preventing dysbiosis (Hamilton et al., 2017; Anhe et al., 2015; Delgado et al., 2014; Kouchaki et al., 2017).

The association between gut microbiota and health has become a hot topic, the rapid progress in this field is ascribed to next generation sequencing methods as well as the ease of maintaining germ free mice (Mardis, 2008; Bhattarai and Kashyap, 2016).

Gut microbes are involved in the biosynthesis of an array of bioactive compounds, contributing to normal human physiological functions or eliciting disease (Fan et al., 2015; Wang et al., 2011). CVD is the leading cause of death worldwide, the association with gut microbiota has been reported in recent few years, which is mediated by gut microbiota derived metabolites (Wang et al., 2011; Tang et al., 2013; Koeth et al., 2013). In this review, we listed gut microbiota derived metabolites and their clinical relevance in cardiovascular health and disease pathogenesis.

## TRIMETHYLAMINE *N* OXIDE (TMAO)

Gut microbiota cleave some trimethylamine containing compounds to produce trimethylamine (TMA), which can be further oxidized as trimethylamine N oxide (TMAO) in the host liver by flavin monooxygenase (FMOs) (Wang et al., 2011; Koeth et al., 2013). FMO3 is the most abundant enzyme in the liver, while FMO1 and FMO2 can also catalyze the oxidation of TMA (Bennett et al., 2013). In some patients with loss-of-function mutation of the FMO3 gene, accumulated TMA in vivo spreads all over the body and is released in sweat and breath, which is a genetic disease named fish odor syndrome (Dolphin et al., 1997; Ulman et al., 2014). The precursors for gut microbiota to produce TMA include TMAO, choline, phosphatidylcholine, carnitine, γ-butyrobetaine, betaine, crotonobetaine and glycerophosphocholine, all of which are abundant in animal diet (Koeth et al., 2013; Wang et al., 2015; Rausch et al., 2013).

**Figure 1:**
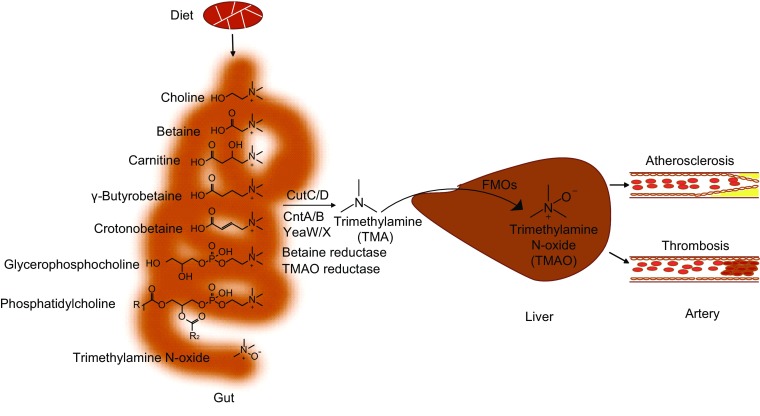
**Metaorganismal pathway of trimethylamine N oxide (TMAO) biosynthesis and linking to cardiovascular disease**. FMOs, Flavin monooxygenases. R1, R2, CH_3_(CH_2_)n_1_(CH=CH)n_2_, n_2_ = 0, 1, 2…..6, n_1_ + 2n_2_  = 15, 17, 19, 21

The diet-gut microbiota-liver to TMAO biosynthesis constitutes a metaorganismal pathway (Fig. 1), including four enzymes involved in production of TMA, choline-TMA lyase (cutC/D) (Craciun et al., 2014), carnitine monooxygenase (cntA/B) (Zhu et al., 2014), betaine reductase (Andreesen, 1994), and TMAO reductase (Pascal et al., 1984). Furthermore, yeaW/X, highly homologous to cntA/B, also contributes to production of TMA. Besides carnitine, yeaW/X can also use choline, γ-butyrobetaine and betaine as substrates to produce TMA (Koeth et al., 2014).

**Figure 2:**
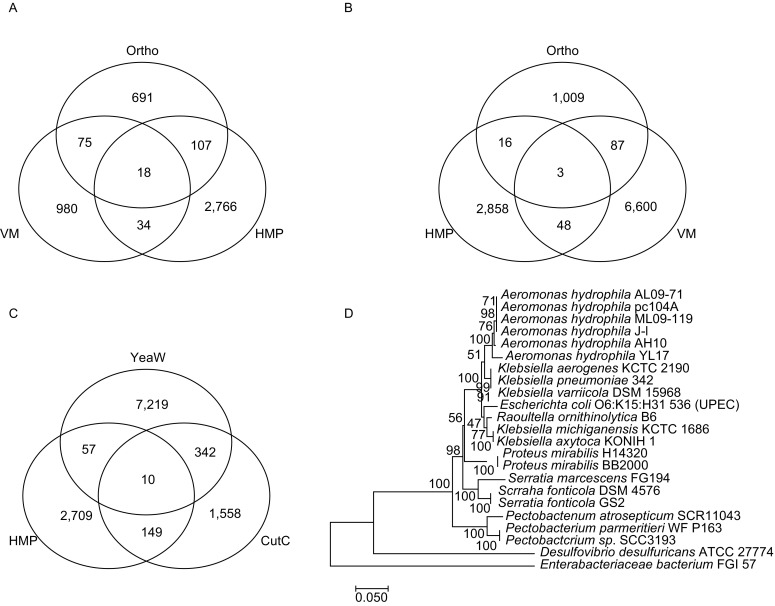
**Predicted bacteria strains encoding the cutC/yeaW/cntA TMA lyases**. (A) Predicted bacteria strains encoding cutC gene. Abbreviation, HMP, the NIH Human Microbiome Project (Data release 1.1, September 26, 2017 e), Ortho, cutC encoding gene of OrthoDB (http://www.orthodb.org/v9.1/) (Zdobnov et al., 2017), and VM, data from the reference (Rath et al., 2017). (B) Predicted bacteria stains encoding yeaW/cntA genes. Ortho, yeaW encoding gene of OrthoDB. (C) Predicted bacterial strains encoding both yeaW and cutC. (D) Phylogenetic gene tree of cutC encoding strains. The Neighbor-Joining tree was built with MEGA7 (Kumar et al., 2016)

CutC/D has been crystalized and the enzymatic mechanism has been demonstrated. CutD, as a radical *S*-adenosylmethionine-activatase, activates CutC, resulting in formation of a glycyl radical. In CutC, the glycyl radical abstracts the hydrogen from cysteine to produce a thiyl radical and further captures the hydrogen atom from choline at C1 position, resulting in molecular rearrangement and TMA production. (Craciun et al., 2014; Kalnins et al., 2015; Bodea et al., 2016). CntA/B is a two-component Rieske-type oxygenase/reductase, carnitine can be first oxidized followed by cleavage at C-N bond by CntA/B to produce TMA and malic semialdehyde (Zhu et al., 2014). Hundreds of bacterial strains are predicted to express cutC/D or cntA/B-yeaW/X in the human gut (Fig. 2A, 2B, 2C and Table S1) (Rath et al., 2017; Martinez-del Campo et al., 2015). *Proteus mirabilis* is a cutC/D expressing bacterium species and since it can grow under both aerobic and anaerobic conditions, it has been used as a model to screen choline trimethylamine lyase inhibitors (Wang et al., 2015). It is most likely the gene tree of cutC substantially differs from species tree, e.g., species of the same genus but with distinct topology for *Klebsiella* (Fig. 2D). FMO3 expression in mice is regulated by sex hormone, repressed by androgens and stimulated by estrogens (Bennett et al., 2013).

Many lines of evidence show the pro-atherogenic property of TMAO. Circulating TMAO level is associated with prevalence of cardiovascular disease and can independently predict incident risk for major adverse cardiac events, including myocardial infarction, stroke or death after adjustment for traditional cardiac risk factors and renal function (Wang et al., 2011; Tang et al., 2013). Circulating choline, betaine and carnitine levels also have been shown associated with prevalence of cardiovascular disease and can predict incident risk for major adverse cardiac events. However, their prognostic values are dependent on the serum TMAO levels (Koeth et al., 2013; Wang et al., 2014). ApoE-null mice fed a chow diet supplemented with TMAO appear to have an enhanced aortic lesion. Furthermore, choline can also increase aortic lesion and promote atherosclerosis but indispensable to gut microbiota, indicating the causal of TMAO in atherosclerosis (Wang et al., 2011). In vitro animal models have also confirmed the prothrombotic effect of TMAO by enhancing platelet aggregation (Zhu et al., 2016). Consistently, oral choline supplementation increases fasting TMAO levels and also enhances platelet aggregation (Zhu et al., 2017).

Mechanisms by which how TMAO can promote atherosclerosis and thrombosis have been studied at the molecular level. TMAO activates vascular smooth muscle cell and endothelial cell MAPK, nuclear factor-κB (NF-κB) signaling, leading to inflammatory gene expression and endothelial cell adhesion of leukocytes (Seldin et al., 2016). Meanwhile, TMAO can also activate the NLRP3 inflammasome (Sun et al., 2016; Boini et al., 2017; Chen et al., 2017). TMAO in vivo can increase scavenger receptor, CD36 and SR-A1 expression, leading to more uptake of modified LDL for macrophage to form foam cell (Wang et al., 2011). On the other hand, TMAO decreases expression of two key enzymes, CYP7A1 and CYP27A1, essential for bile acid biosynthesis and multiple bile acid transporters (OATP1, OATP4, MRP2 and NTCP) in the liver, which decreases bile acid pool, resulting in decreased reverse cholesterol efflux (Koeth et al., 2013). Moreover, TMAO increases endoplasmic recticulum calcium release in platelet cell, consequently leading to platelet aggregation and thrombosis (Zhu et al., 2016).

The association between TMAO and cardiovascular disease has been highlighted in different groups by using different cohorts worldwide (Troseid et al., 2015; Suzuki et al., 2016, 2017; Schuett et al., 2017). Besides cardiovascular disease, TMAO also contributes to renal insufficiency and mortality risk in chronic kidney disease, type II diabetes, insulin resistance, non-alcoholic fatty liver disease and colorectal cancer as well (Tang et al., 2015; Shan et al., 2017; Oellgaard et al., 2017; Kummen et al., 2017). These studies indicate circulating TMAO levels has the potential to be managed for TMAO related diseases intervention. Specially, targeting the metaorganismal pathway for TMAO biosynthesis can be achieved by a few key steps, including inhibiting gut microbiota cleavage of TMA containing compounds in nutrient via enzymatic inhibitor, controlling intake of diet rich in TMA precursors and inhibiting the oxidation of TMA to TMAO.

As expected, the injection of antisense oligonucleotide to *Ldlr*-null mice decreases the hepatic *Fmo3* gene expression, resulting in decreased mouse plasma TMAO thereby decreasing aortic lesion in western diet fed mice (Shih et al., 2015). However, the accumulated TMA in mice will show fish odor syndrome. In addition, *Fmo3* knockdown exacerbates hepatic endoplasmic reticulum (ER) stress and inflammation (Warrier et al., 2015). Thus, developing gut microbiota enzymatic inhibitors to inhibit TMA formation will be more practical.

A choline analogue, 3,3-dimethylbutanol (DMB), has been uncovered with inhibitory effect to choline TMA lyase activity in turn decreasing circulating TMAO, and therefore attenuating the promoting role of choline in atherosclerosis (Wang et al., 2015). DMB is a natural product, distributed in certain balsamic vinegars, red wines, cold-pressed extra virgin olive oils and grapeseed oils. DMB has not been found any adverse effect to the liver or renal functions even as high as in mice drinking water up to 1% (Wang et al., 2015). Very recently, we have found that several more choline analogues show more potent in inhibiting choline TMA lyase activity than DMB (to be published). But inhibitors to different enzymatic cleavage of other substrates are still needed. Furthermore, a study shows that resveratrol, a phytoalexin, can decrease plasma TMAO and subsequent atherosclerosis in ApoE^−/−^ mice via gut microbiota remodeling, characterized by increased levels of the genera *Lactobacillus* and *Bifidobacterium* with increased bile salt hydrolase activity to increase bile acid neosynthesis, suggesting the potential of resveratrol as prebiotics (Chen et al., 2016).

## UREMIC TOXINS

**Figure 3:**
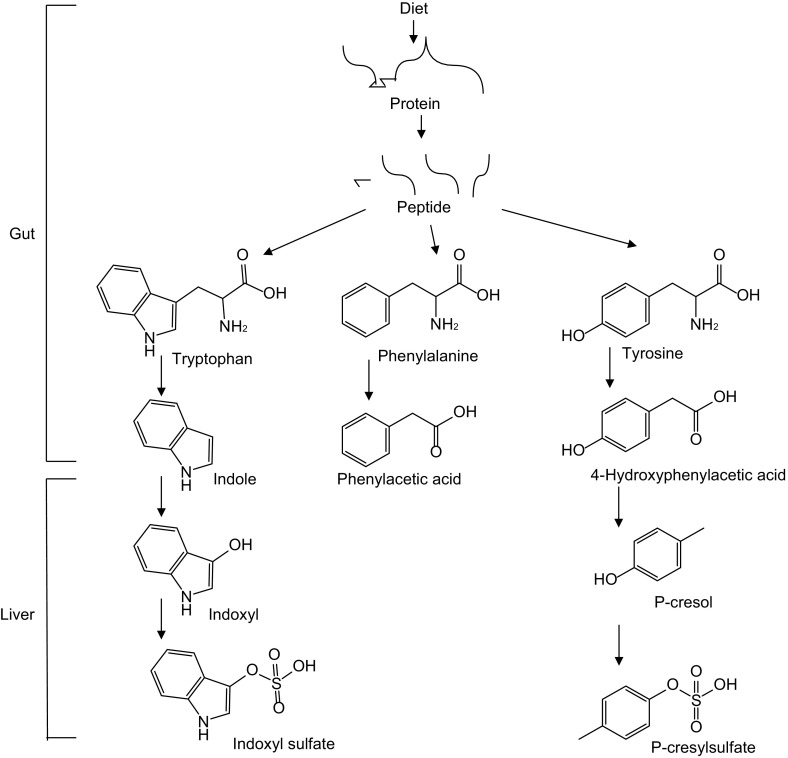
**Gut microbiota involved in the biosynthesis of phenylacetic acid, p-cresyl sulfate and indoxyl sulfate**

Toxins, such as urea and asymmetric dimethylarginine, can be accumulated in blood during chronic kidney disease (CKD), associated to CKD complications especially heart failure which is the leading cause of CKD mortality (Glassock 2008). Moreover, protein-bound uremic toxins such as indoxyl sulfate, indoxyl glucuronide, indoleacetic acid, *p*-cresyl sulfate, *p*-cresyl glucuronide, phenyl sulfate, phenyl glucuronide, phenylacetic acid and hippuric acid have been reported to be increased in serum in hemodialysis patients (Itoh et al., 2013). These uremic toxins are gut microbiota derived metabolites of amino acids (Devlin et al., 2016). The aromatic amino acids in proteins, phenylalanine, tyrosine and tryptophan, can be metabolized by gut microbiota (Nallu et al., 2017; Pereira-Fantini et al., 2017). Both microbiota and host liver are involved in biosynthesis of these uremic toxins (Fig. 3) (Devlin et al., 2016; Meyer and Hostetter 2012; Webster et al., 1976; Gryp et al., 2005).

The serum indoxyl sulfate level, positively correlated with coronary atherosclerosis scores, might be a predicative mechanistic biomarker of coronary artery disease severity (Hsu et al., 2013). Further studies have shown that indoxyl sulfate aggravates cardiac fibrosis, cardiomyocyte hypertrophy and atrial fibrillation (Yisireyili et al., 2013; Aoki et al., 2015). Atrial fibrillation, the most common clinical arrhythmia, results in cardiovascular morbidity and mortality attributed to congestive heart failure and stroke (Hung et al., 2017). Mechanistically, indoxyl sulfate enhances platelet activities, increases response to collagen and thrombin, leading to thrombosis (Yang et al., 2017). Vascular smooth muscle cell calcification is associated with major adverse cardiovascular events while indoxyl sulfate has been found to promote vascular smooth muscle cell calcification (Zhang et al., 2018). Indoxyl sulfate activates NF-κB signaling pathway, leading to increased intercellular adhesion molecule-1 (ICAM-1) and monocyte chemotactic protein-1 (MCP-1) expression in endothelial cells (Tumur et al., 2010). ICAMs over-expression in endothelial cells is the initiating step for atherosclerotic plaque formation (Moss and Ramji 2016). Indoxyl sulfate inhibits nitric oxide production and induces reactive oxygen species production, gradually damaging endothelial cell layer (Tumur and Niwa 2009). Taken together, these studies indicate indoxyl sulfate mechanistically linked to CVD at the molecular and cellular levels.

*p*-Cresyl sulfate is a biomarker in predicting cardiovascular event and renal function progression in CKD patients without dialysis (Lin et al., 2014; Wu et al., 2012). *p*-Cresyl sulfate can induce NADPH oxidase activity to produce reactive oxygen species, resulting in cardiomyocyte apoptosis and subsequent diastolic dysfunction (Han et al., 2015). Apocynin and N-acetylcysteine, inhibitors to NADPH oxidase, can attenuate the effect of p-cresyl sulfate induced apoptosis (Han et al., 2015). *p*-Cresyl sulfate increased endothelial cell tumor necrosis factor-α (TNF-α), MCP-1, ICAM and VCAM expression, therefore mechanistically promotes atherogenesis (Jing et al., 2016). Given that p-cresyl sulfate, very similar to indoxyl sulfate, is notoriously difficult to eliminate by dialysis (Gryp et al., 2005), it is most likely that intervening the biosynthesis pathway is the best way to attenuate such toxic effect.

## SHORT CHAIN FATTY ACIDS

Short chain fatty acids (SCFAs) refer to fatty acids with a carbon number of not greater than 6, including three major SCFAs, acetic acid, propionic acid, butyric acid, and two less abundant valeric acid and caproic acid. Acetic acid, the most abundant SCFA in the colon with more than half of the total SCFA detected in feces, can be generated by carbohydrate fermentation, or synthesized from hydrogen and carbon dioxide or formic acid through the Wood-Ljungdahl pathway (Miller and Wolin, 1996; Louis et al., 2014). Three distinct pathways including succinate pathway, acrylate pathway, and propanodiol pathway, can generate propionic acid (Reichardt et al., 2014). Butyric acid-producing bacteria use two different pathways, the pathway using phosphotransbutyrylase and butyrate kinase enzymes to convert butyryl-CoA into butyrate (e.g., Coprococcus species) (Louis et al., 2004; Flint et al., 2015), and the butyryl-CoA/acetate CoA-transferase pathway, in which butyryl-CoA is converted to butyric acid in a single step enzymatic reaction (e.g., Faecalibacterium, Eubacterium and Roseburia) (Louis et al., 2010).

The proposed biosynthesis of SCFAs in bacteria is sequential from glycolysis of glucose to pyruvate, to acetyl-coA, and eventually to acetic acid, propionic acid and butyric acid. Intriguingly, amino acids are alternative substrate for SCFAs biosynthesis. Glucose and amino acids can be digested from starch and protein in small intestine, respectively. Glucose and amino acids can be absorbed into circulating system rapidly prior to reaching colon where microbes accumulated, and the main substrate for the microbes to produce SCFAs is dietary fiber. Both inulin, a kind of fructan, found in many plants, and guar gum are prebiotic fiber (den Besten et al., 2015, 2014; Boets et al., 2015). The beneficial effect of inulin include increasing calcium absorption in colon and decreasing food intake thereafter loss-of-weight (Abrams et al., 2007; Harrold et al., 2013; Liber and Szajewska 2013). Many clinical trials have confirmed a lot of benefits of inulin on health promoting functions and reducing the risk of many diseases, leading to inulin extensively used as nutrient supplement (Kaur and Gupta 2002). Germ free animals have trace amounts of SCFAs, possibly from diet (Hoverstad et al., 1985; Hoverstad and Midtvedt 1986).

Acetic acid producing bacteria are included in Acetobacteraceae containing 10 genera which can oxidize sugars or ethanol to produce acetic acid during fermentation (Raspor and Goranovic 2008). At least 33 strains can produce propionic acid and 225 strains can produce butyric acid by fermenting dietary fiber in human gut (Reichardt et al., 2014; Vital et al., 2014). More interestingly, dietary fiber can selectively increase SCFAs producing bacterium abundance (Zhao et al., 2018).

Short chain fatty acids play important roles in human health. SCFAs can be used to feed colonocyte, maintain gut barrier and inhibit pathogenic microbe proliferation due to acidic pH condition (Hashemi et al., 2017; Cherrington et al., 1991; Prohaszka et al., 1990; Duncan et al., 2009; Manrique Vergara and Gonzalez Sanchez, 2017). SCFAs can work as inhibitors to histone deacetylase (HDAC), which decreases expression of the miR-106b family and increases p21 expression, leading to human colon cancer cell apoptosis (Chen et al., 2003; Hu et al., 2011; Heerdt et al., 1997). SCFAs functions as anticancer therapeutics (Chen et al., 2003). There are three SCFAs receptors expressed in colon epithelial cells including GPR43 (FFAR2), GPR41 (FFAR3) and GPR109A (Karaki et al., 2008; Tazoe et al., 2009; Ahmed et al., 2009). These receptor can trigger secretion of the incretin hormone glucagon-like peptide (GLP)-1 to influence metabolic state and increase peripheral glucose clearance (den Besten et al., 2015; Tolhurst et al., 2012). GPR109A can only be activated by butyric acid, not by acetic acid or propionic acid (Ahmed et al., 2009). Meanwhile, there is another SCFA receptor, OLFR78, expressed in blood vessel and activated by acetic acid and propionic acid but not by butyric acid involved in the modulation of the blood pressure (Pluznick et al., 2013; Pluznick 2014). In addition, recent studies have found a panel of SCFA receptors expressed in distinct cell types, e.g., FFAR2 and FFAR3 in pancreatic β-cells, FFA3 in neurons, FFA2 in leukocytes, as well as FFA2 and GPR109A in adipocytes, indicating that the ubiquitous and cell-type specific functions of SCFAs (Ahmed et al., 2009; Nilsson et al., 2003). Thus, gut microbiota derived SCFAs actively participate in the host energy hemostasis regulation, play critical regulatory functions in brain, muscle, airway, white adipose tissue, brown adipose tissue and blood vessel physiology (Kasubuchi et al., 2015).

A double-blind randomized placebo-controlled cross-sectional study, where eleven normotensive subjects with no family history of essential hypertension were recruited, has found supplementation of miglyol rich in caprylic (8:0) and capric acids (10:0) results in decreased diastolic blood pressure (MacIver et al., 1990). Furthermore, rodent model studies have shown that SCFAs administration can decrease systolic blood pressure mediated by GPR41 expressed in vascular endothelium, while GPR41 knock out mice have isolated systolic hypertension compared with wild-type (WT) mice (Natarajan et al., 2016). Olfr78, a member of the G-protein-coupled receptor family expressed in vascular smooth muscle cells, contributes to blood pressure control as Olfr78-deficient mice showed hypertension (Miyamoto et al., 2016). Therefore, such causality studies including randomized controlled trial and instrumental rodent genetics model, have conclusively shown the pivotal role of SCFAs in blood pressure regulations.

## PHYTOESTROGENS

**Figure 4:**
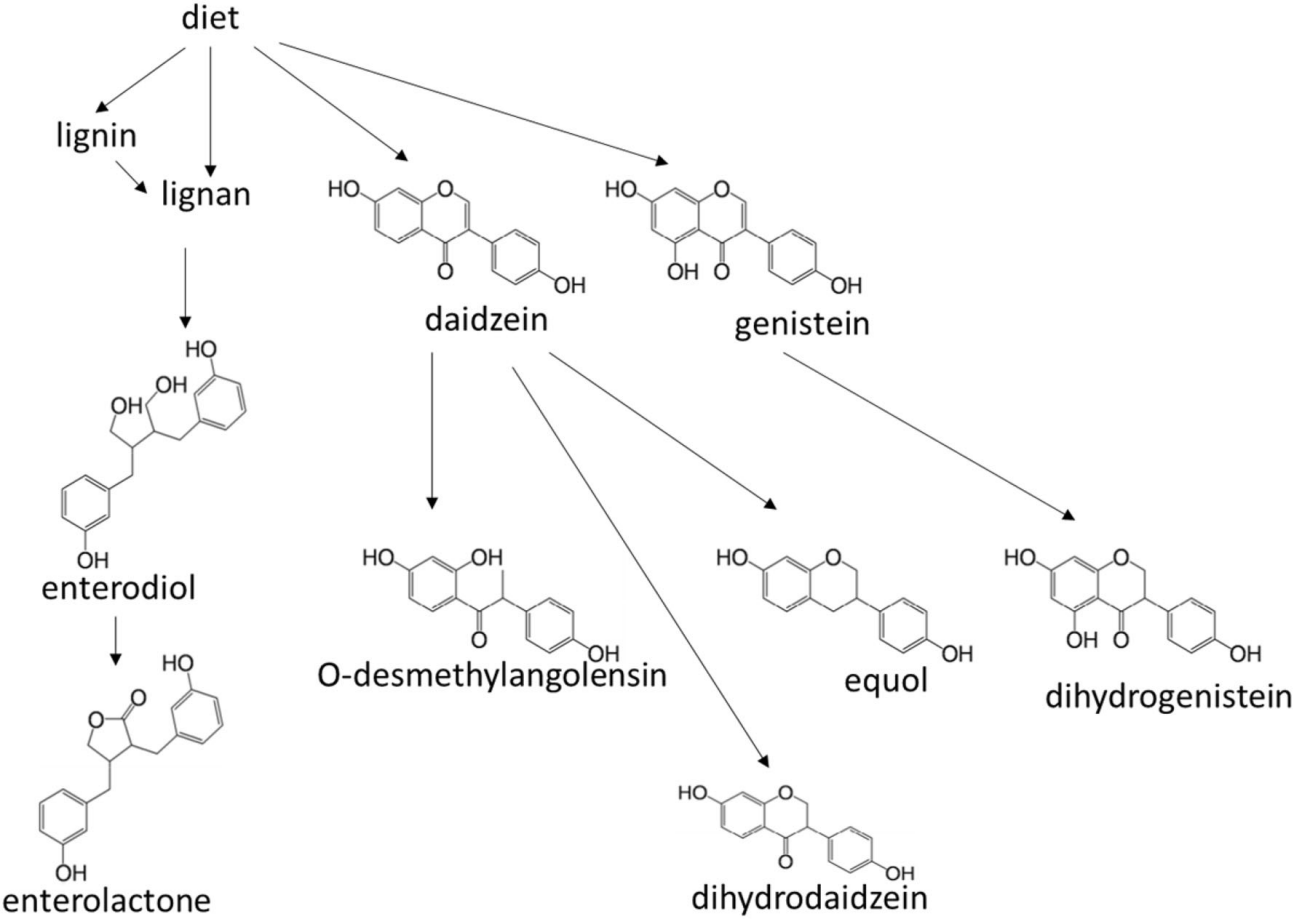
**Structural formulas of phytoesterogens and the metabolism pathways**

Phytoestrogens in plant can protect itself from attack by modulation of the fertility of plant predators, vertebrate herbivores (Hughes, 1988). Phytoestrogens are similar to human estrogens in structure. There are three main groups of phytoestrogens, isoflavones, ellagitannins and lignans (Gaya et al., 2108). In the gut, phytoestrogens can be further metabolized to more active molecules, such as equol, *O*-desmethylangolensin (*O*-DMA), dihydrodaidzein, dihydrogenistein, enterolactone and enterodiol (Fig. 4) (Gaya et al., 2108; Axelson and Setchell 1981; Wang et al., 2005). The biosynthesis pathway of enterolactone and enterodiol have been found from several bacterium strains metabolizing lignan (Vanharanta et al., 2003). Both pinoresinol and lariciresinol, precursors of enterolactone and enterodiol, are a structural moiety in lignin. Lignin is an abundant plant-derived polymer secondary to cellulose in amount in the earth (Vanharanta et al., 2003). Lignin can be degraded by gut microbiota to release lignans (DeAngelis et al., 2011). Equol and O-DMA can be metabolized from daidzein in the gut by several bacterium strains, such as *Adlercreutzia equolifaciens*, *Eggerthella sp*. YY7918, *Lactococcus garvieae*, *Slackia equolifaciens*, *Slackia isoflavoniconvertens*, *Slackia sp*. NATTS (Braune and Blaut, 2018; Guadamuro et al., 2017; Matthies et al., 2012; Frankenfeld et al., 2014).

Phytoestrogens are reported to reduce breast cancer for postmenopausal women (Goodman et al., 2009). In animal model, pretreatment of phytoestrogen-rich, *Pueraria mirifica* tuberous powder resulted in decreasing the virulence of rat breast tumor development induced by 7,12-dimethylbenz(a)anthracene (Cherdshewasart et al., 2007). Besides breast cancer, phytoestrogens may have protective action against prostate, bowel and other cancers, cardiovascular disease, brain function disorders and osteoporosis (Zhang et al., 2016; Ward and Kuhnle 2010; Arbabi et al., 2016; Menze et al., 2015; Trieu and Uckun 1999; Chiechi et al., 1999; Wang et al., 2011; Zhang et al., 2004; Lephart et al., 2001). However, a few investigations implicate that the controversial role of phytoestrogens including increasing colorectal cancer and prostate cancer risk and indicate little supportive evidence of phytoestrogens decreasing cardiovascular disease risk (Ward et al., 2010; van der Schouw et al., 2005; Peterson et al., 2010).

Enterolactone is a biphenol, which can function as anti-oxidant. A study shows that high serum enterolactone level is associated with reduced CVD mortality (Vanharanta et al., 2003). Furthermore, low serum enterolactone is associated with increased in vivo lipid peroxidation, assessed by plasma F2-isoprostane concentrations (Vanharanta et al., 2002). In addition, urinary total and individual phytoestrogens were significantly inversely associated with serum C-reactive protein (CRP; an inflammation biomarker) (Reger et al., 2017). Phytoestrogens can bind to estrogen receptors (Morito et al., 2001), which either mimics estrogen or works as antagonist (Fitzpatrick, 1999). Thus, the effects of phytoestrogens can be biphasic: for example, phytoestrogens both increases vasodilation and nitric oxide metabolism that may have a favorable impact on vascular health; on the other hand, phytoestrogen may also have some prothrombotic or proinflammatory effects that may offset other benefits (Herrington, 2000). Both enterolactone and enterodiol can alleviate the effect of peripheral blood lymphocytes activated by lipopolysaccharide (Corsini et al., 2010). Such lymphocytes activation leads to inhibitory-κB (I-κB) degradation and nuclear factor-κB (NF-κB) activation thereby resulting in TNF-α production (Corsini et al., 2010). Thus, both enterolactone and enterodiol may have pro-anti-inflammatory role.

## ANTHOCYANINS

Anthocyanins are glycosyl-anthocyanidins, widely distributed in plant vacuole with pH depending color. Anthocyanidins are flavones with different functional groups covalently linked to the three cycles. Anthocyanins have been found with beneficial effects on obesity and diabetes control, cardiovascular disease and cancer prevention, and visual and brain function improvement (Tsuda, 2012; Hannum, 2004). Mechanistically, the beneficial effect of anthocyanins on cardiovascular health include working as an antiplatelet agent in atherosclerosis and other CVD prevention, inducing nitric oxide formation in vessel thereby enhancing vasorelaxation, protecting cardiac cells from oxidative-stress-induced apoptosis, and increasing HDL cholesterol as well (Gaiz et al., 2018; Stoclet et al., 1999; Hassellund et al., 2013; Isaak et al., 2017).

**Figure 5:**
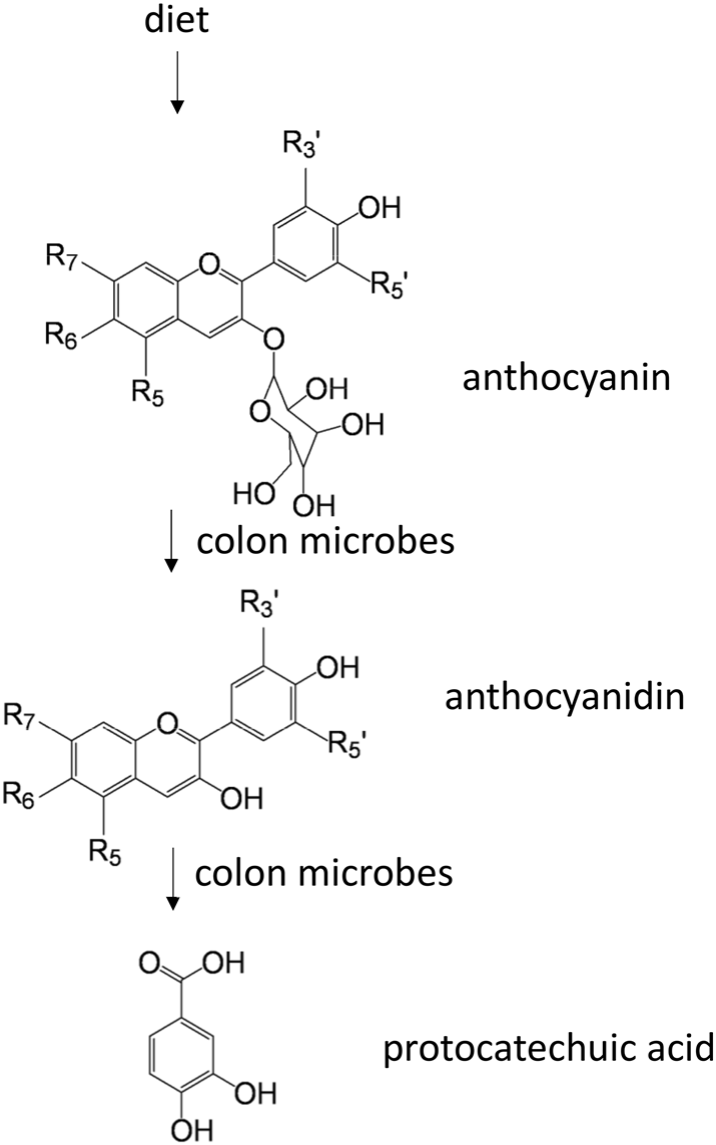
**Colon microbes contribute to protocatechuic acid biosynthesis from diet anthocyanins**. R_3_′ = H, OH or OCH_3_; R_5_′ = H, OH or OCH_3_; R_5_ = OH or OCH_3_; R_6_ = H or OH; R_7_ = OH or OCH_3_. R_5_, R_7_ can be glycosylated if it is a hydroxyl group

Further investigations have confirmed that the beneficial effect of some anthocyanins on atherosclerosis is mediated by gut microbiota metabolites. Ingested dietary anthocyanins are absorbed with a small part while large amounts are likely to enter the colon to be degraded by gut microbiota as free anthocyanidins and protocatechuic acid (PCA) (Fig. 5) (Aura et al., 2005). Anthocyanidin-3-glucoside promotes reverse cholesterol transport mediated by its gut microbiota metabolite, PCA. PCA can reduce macrophage miR-10b expression, therefore increasing ABCA1 and ABCG1 expression (Wang et al., 2012). Gallic acid (GA), one of the microbiota anthocyanin metabolites, has been shown increasing nitric oxide (NO) levels by increasing phosphorylation of endothelial nitric oxide synthase (eNOS) (Radtke et al., 2004). GA inhibited angiotensin-I converting enzyme (ACE), leading to reduced blood pressure in spontaneously hypertensive rats (SHR) comparable to captopril (Kang et al., 2015). These results suggest that GA isolated from *Spirogyra sp*. exerts multiple therapeutic effects and has a great potential for CVD intervention.

Anthocyanins can also modulate gut microbiota community structure. For example, malvidin-3-glucoside can enhance the growth of some beneficial bacterium such as *Bifidobaterium* spp. and *Lactobacillus* spp. (Hidalgo et al., 2012). On the other hand, gallic acid, one of the microbiota anthocyanin metabolites, can reduce some potentially harmful bacteria such as *Clostridium histolyticum*, without negative effect on beneficial bacteria (Hidalgo et al., 2012). Study on comparison in gut microbiota fingerprints between cardiovascular disease patients and healthy controls has shown that the diversity of beneficial bacteria was reduced in patients with cardiovascular disease (Vamanu et al., 2016). Thus, anthocyanins play critical role in shaping the microbiota taxonomic composition especially under CVD conditions.

## BILE ACIDS

**Figure 6:**
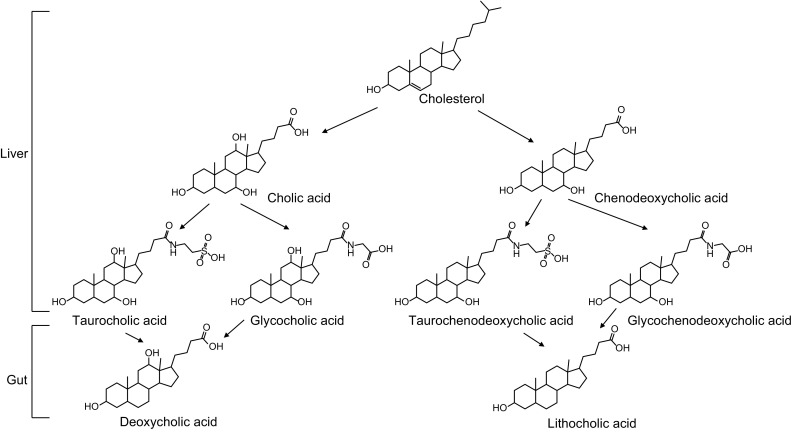
**The main bile acids and their metabolic pathways**

Bile acids are synthesized from cholesterol in liver. The initial products are chenodeoxycholic acid (CDCA) and cholic acid (CA) (Fig. 6), and then conjugated with glycine or taurine, stored and concentrated in gallbladder (Wahlstrom et al., 2016; LaRusso et al., 1974). Bile acids produced in liver are called as primary bile acids. Bile acids are released into duodenum after meal to emulsify dietary fats and oils for digestion and help absorb lipid soluble vitamins (Danielsson, 1963; Hollander et al., 1977; Barnard and Heaton, 1973; Miettinen, 1971). In ileum, conjugated bile acids are then reabsorbed and carried in the portal blood to liver. This process is called enterohepatic circulation and preserves more than 95% of the bile acid pool (Wahlstrom et al., 2016). In distal ileum, conjugated bile acids are hydrolyzed to remove glycine or taurine by bile salt hydrolase in microbes to escape reuptake by apical sodium dependent bile acid transporter and dehydroxylated by microbes as deoxycholic acid or lithocholic acid, which are called as secondary bile acids (Fig. 6**)**, (Wahlstrom et al., 2016; Chiang, 2009). The deconjugated bile acids are hydrophobic and it can be excreted as feces, which constitutes the last step of reverse cholesterol efflux to decrease circulating cholesterol (Dawson and Karpen, 2015), therefore the risk for atherosclerosis can be decreased.

Bile acid can modulate gut microbiota composition by killing bacterium in a species and dosage dependent way (Yokota et al., 2012). Bile acids are associated with metabolic disease, obesity, diarrhea, inflammatory bowel disease, colorectal cancer and hepatocellular carcinoma as well (Joyce and Gahan, 2016).

Bile acids can work as hormone to act on farnesoid X receptor (FXR) and G protein-coupled membrane receptor 5 (TGR5) to decrease triglyceride accumulation, fatty acid oxidation, decrease the expression of pro-inflammatory cytokines and chemokines in aorta through the inactivation of NF-κB (Levi, 2016; Porez et al., 2012).

Gut microbiota can affect cardiovascular health via secondary bile acids, deoxycholic acid and lithocholic acid, both of which are the main ligand for TGR5 (Fiorucci et al., 2010; Duboc et al., 2014). Primary bile acids including chenodeoxycholic acid and cholic acid, with FXR as their the receptor, have distinct effects on cardiac health when compared to secondary bile acids (Fiorucci et al., 2010). Consistently, the serum level of primary bile acids were found decreased while ratios of secondary bile acids to primary bile acids were increased in cardiovascular disease patients compared to healthy controls (Mayerhofer et al., 2017).

## LIPOPOLYSACCHARIDE

Distinguished from the abovementioned gut microbiota derived metabolites, lipopolysaccharide (LPS, also called as endotoxin) is a component of outer-membrane of Gram-negative bacteria with a very complicated structural formula composed of lipid and saccharide. LPS is released from the bacterial membrane after destruction with the capacity of inducing systemic inflammation and sepsis (Beutler and Rietschel, 2003). For healthy subjects, gut-blood barrier prevents LPS entering circulating blood. However, the gut-blood barrier leak due to dysbiosis results in bacterium entering the bloodstream. For the periodontal patients, bacterium can directly enter circulating blood, leading to increased levels of circulating LPS (Fukui et al., 1991; Wang et al., 2015; de Punder and Pruimboom, 2015; Lakio et al., 2006).

LPS can induce foam cell formation and cholesteryl ester accumulation from native low density lipoprotein, indicating LPS is proatherogenic (Lakio et al., 2006; Funk et al., 1993). LPS induces CD14 and SR-AI expression in macrophages via JNK1, leading to oxLDL uptake and foam cell formation (An et al., 2017). LPS binding protein (LBP) is synthesized in liver and released to circulating blood (Schumann et al., 1990). Serum LBP level in patients with angiographically confirmed coronary artery disease (CAD) found significantly higher than controls without CAD is an independent predictive biomarker for total and cardiovascular mortality (Lepper et al., 2011). Moreover, the high affinity binding complex of LPS-LBP binds to monocyte and macrophage, triggering the secretion of tumor necrosis factor (Schumann et al., 1990). Toll-like receptor 4 (TLR4) is the membrane receptor of LPS, when activated, triggering NF-κB signaling and producing proinflammatory cytokines (Lu et al., 2008). Further, inflammatory caspase-4, -5 and -11 directly recognize bacterial LPS, both of which trigger pyroptosis (Shi et al., 2015). Low serum selenium or selenoprotein P (SePP) levels have been repetitively observed in severe sepsis, and both purified SePP and synthetic peptides corresponding to the His-rich motifs neutralized LPS (Zhao et al., 2016). Very recently, a study shows itaconate is required for the activation of the anti-inflammatory transcription factor Nrf2 (also known as NFE2L2) by lipopolysaccharide in mouse and human macrophages via dicarboxylation of KEAP1 (Mills et al., 2018). Taken together, LPS is a mechanistic biomarker for CAD.

## PROSPECT

More and more gut microbiota derived metabolites have been unveiled as crucial factor contributing to cardiovascular health and disease. Thus, a better understanding of the gut microbe pathways involved in the biosynthesis of CVD related metabolites would greatly facilitate managing cardiac health especially preventing CVD.

Apparently, for mechanistic biomarker discovery and CVD management, it is of primary importance to pinpoint the causal role of gut microbiota derived metabolites. Koch’s postulate, which states that a given pathogen leads to a distinct disease, have been evolving into molecular and ecological Koch’s postulate including CVD (Vonaesch et al., 2018). Therefore, many ongoing efforts have been focusing on the causality of gut microbiota derived metabolites in CVD. Key methodologies include randomized controlled trials (Tang et al., 2013; Panigrahi et al., 2017), Mendelian randomization approach (Mendelson et al., 2017) and gnotobiotic animal models (Hibberd et al., 2017).

Given that diet is the most important factor shaping the dynamics of gut microbiotia (Rothschild et al., 2018), integrative studies on diet shaped microbiota-host interactions have the potential to offer us novel insight on CVD mechanisms. From the microbiota side, there is big room to study molecular genetics mechanisms by which how the physiology and pathology relevant microbiota taxonomic and functional profiles are regulated. Of note, studies on the immune mechanisms of CVD allow us to connect gut microbiota derived metabolites to key immune components of distinct immune cell and cytokine profile dynamics. We envision discovering predicative mechanistic CVD microbiome biomarkers and exploiting the probiotics and prebiotics therapeutics continue to be of primary priority.

## Electronic supplementary material

Below is the link to the electronic supplementary material.

Supplementary material 1 (XLSX 15 kb)
